# miR-221-3p Delivered by BMMSC-Derived Microvesicles Promotes the Development of Acute Myelocytic Leukemia

**DOI:** 10.3389/fbioe.2020.00081

**Published:** 2020-02-14

**Authors:** Xuewu Zhang, Yu Xu, Jinghan Wang, Shuqi Zhao, Jianhu Li, Xin Huang, Huan Xu, Xiang Zhang, Shanshan Suo, Yunfei Lv, Yi Zhang, Wenjuan Yu

**Affiliations:** Department of Hematology, Zhejiang University School of Medicine First Affiliated Hospital, Hangzhou, China

**Keywords:** BMMSC, microvesicles, miR-221-3p, AML, cell proliferation and invasion, cell cycle

## Abstract

**Objective:** The study aims to investigate the effects of miR-221-3p in bone marrow mesenchymal stem cell (BMMSC)-derived microvesicles (MVs) on cell cycle, proliferation and invasion of acute myelocytic leukemia (AML).

**Methods:** Bioinformatics was used to predict differentially expressed miRNAs (DEmiRNAs) in AML. The morphology of BMMSC-derived MVs was observed under an electron microscope, and the positional relation of MVs and OCI-AML2 cells was observed by a fluorescence microscope. MTT, Transwell, and flow cytometry assays were used to analyze the effects of MVs on OCI-AML2 cells. The targeted relationship between miR-221-3p and CDKN1C was detected by dual luciferase assay.

**Results:** It was verified that miR-221-3p promoted the proliferation, invasion and migration of OCI-AML2 cells, and induced the cell cycle arrest in G1/S phase as well as inhibited cell apoptosis. Further studies showed that MVs promoted the proliferation, migration and invasion of AML, and induced the cell cycle arrest in G1/S phase through miR-221-3p. It was confirmed that miR-221-3p can directly target CDKN1C to regulate cell cycle, proliferation and invasion of AML.

**Conclusion:** miR-221-3p in BMMSC-derived MVs regulated AML cell cycle, cell proliferation and invasion through targeting CDKN1C. miR-221-3p and CDKN1C were considered to be potential targets and biomarkers for the treatment of AML in clinic.

## Introduction

Acute myelocytic leukemia (AML) is a malignant tumor of abnormal clonal in immature myeloid hematopoietic cells with high heterogeneity, which is characterized by differentiation and maturation disorders along with block of apoptosis in clonal hematopoietic stem cells or progenitor cells, leading to malignant proliferation and accumulation of cells in the bone marrow, thus affecting normal hematopoiesis (Coombs et al., 2016; Khwaja et al., 2016). AML is the most-common acute leukemia in adults, but it predominantly occurs in older people (>60 years of age), with a median age at diagnosis of 67 (Coombs et al., 2016). It typically presents with a rapid onset of symptoms that are attributable to bone marrow failure and may be fatal within weeks or months when left untreated. Currently, chemotherapy and hematopoietic stem cell transplantation are the main treatments, but the success rate of AML cure remains low (Cornelissen and Blaise, 2016; Stein and Tallman, 2016). Therefore, it is particularly important to study the pathogenesis of AML and explore the possible therapeutic approaches.

Microvesicles (MVs) are extracellular vesicles between 100 nm and 1 μm that derived from normal cells and cancer cells. MVs can transfer proteins, glycoproteins, lipids, nucleic acids, and cytokines from maternal cells to recipient cells, promoting phenotype changes of recipient cells and playing an important role in intercellular communication (Hansen et al., 2014; Gopal et al., 2017; Abbasian et al., 2018). Studies have found that tumor-derived MVs (TMV) can interact directly with tumor cells and play a macro-messenger role to promote the transfer of molecular substances between tumor cells to facilitate tumor growth (Stec et al., 2015a,b). MVs derived from bone marrow mesenchymal stem cell (BMMSC) can promote tumorigenesis and development (Crompot et al., 2017; Boyiadzis and Whiteside, 2018). miRNAs in MVs, as post-transcriptional regulatory elements, directly regulate gene expression, target mRNA expression and translation or induce mRNA degradation to reduce protein synthesis by directly binding with the 3′-untranslation region (3′-UTR) of specific mRNA targets (Braicu et al., 2015; Jerez et al., 2019), ultimately induce multiple pathophysiological processes, such as leukemia stem cell formation, regulation of tumor cell proliferation, angiogenesis, invasion, metastasis, and immune escape to modulate leukemia development (Braicu et al., 2015; Del Principe et al., 2017).

microRNAs (miRNA) are a class of evolutionarily conserved 22 to 24-nucleotide small RNAs in length, which are widely found in eukaryotic cells with molecular functions to regulate cell differentiation, proliferation and apoptosis (Summerer et al., 2013; Zheng et al., 2013). miR-221-3p has important regulatory effects on a variety of cancers as an important miRNA. Studies have reported that in cervical squamous cell carcinoma, miR-221-3p in MVs promotes lymph angiogenesis and lymphatic metastasis by targeting VASH1 (Zhou et al., 2019), and promotes angiogenesis by targeting THBS2 (Wu et al., 2019). We previously found that miR-221-3p was significantly highly expressed in AML patients through bioinformatics, and miR-221-3p mainly existed in BMMSC-derived MVs. These results suggest that miR-221-3p in BMMSC-derived MVs has certain regulatory effects on AML cells.

Therefore, in this paper, we explored the regulatory effects and mechanism of miR-221-3p in BMMSC-derived MVs on cell cycle, cell proliferation and invasion of AML through *in vitro* experiments, so as to further understand the pathogenesis of AML and provide new ideas for future clinical diagnosis and treatment.

## Materials and Methods

### Cell Lines and Patients

Normal human BMMSCs were purchased from Kunming cell bank, Chinese Academy of Sciences (No. 3153C0001000000244). BMMSCs were isolated from AML patients and human AML cells OCI-AML2 (BNCC341618) were purchased from BeNa Culture Collection (China).

Fifteen AML patients and 18 control samples (peripheral blood or bone marrow) were obtained with the informed consent of the patient or healthy subject and were collected at the First Affiliated Hospital of Zhejiang University through the protocol approved by the review committee.

### Bioinformatics Analysis

AML-related miRNA expression dataset GSE49665 was obtained from GEO database (https://www.ncbi.nlm.nih.gov/geoprofiles/) to screen differentially expressed miRNAs (DEmiRNAs) and determine target miRNAs. Target miRNAs were found to be highly expressed in the MVs of fiber cells and mesenchymal stem cells (MSCs) via searching expression location In the EV miRNA database (http://bioinfo.life.hust.edu.cn/EVmiRNA). The downstream target genes of the target miRNAs were predicted by TargetScan database (http://www.targetscan.org/vert_72/), miRSearch database (https://www.exiqon.com/miRSearch), and mirDIP database (http://ophid.utoronto.ca/mirDIP/index.jsp), and differential analysis was conducted on AML gene expression in TCGA. The down-regulated genes in AML were selected to intersect with the predicted downstream target genes. Finally, the target genes with the most significant expression changes were detected by signaling pathway enrichment analysis.

### Isolation, Culture and Analysis of BMMSC

BMMSCs were obtained by density gradient centrifugation. The bone marrow fluids were centrifuged at 1,000 rpm for 10 min, while the lipids and supernatant were absorbed and discarded. The remaining marrow fluids were added with equal quantity of PBS buffer and mixtured, centrifuged at 1,000 rpm for 10 min, and the supernatant was discarded. Then cell suspensions were prepared with 2 mL PBS buffer at a density of 4 × 10^7^ cells, carefully superimposed on 5 mL Percoll separation solution (at a density of 1.077 g/mL), and centrifuged at 2,300 rpm for 30 min. After centrifugation, the liquids from top to bottom are: platelet and plasma diluent layer, yellow-brown annular cloud-like mononuclear cell layer, lymphocyte separation liquid layer, red blood cells and granulocyte layer. The mononuclear cell layer was absorbed and mixed with PBS buffer at a ratio of 1:2, and then centrifuged at 1,500 rpm for 10 min. All centrifugations were carried out at room temperature. The supernatant was discarded and cells were washed twice. 1 × 10^6^ cells/mL were inoculated in a 25 cm^2^ culture bottle with 5 mL BMMSCs medium (containing 10% fetal bovine serum, FBS). After 2–3 days, non-adhesive cells were removed, and monolayer adherent cells were spread to 70–80% of the bottom of the culture bottle. Cells were then isolated in a trypsin solution (0.25% trypsin/0.1% EDTA PBS solution, free of magnesium/magnesium and phenolic red) (Aurogene, Rome, Italy) and re-inoculated at a density of 3.5 × 10^3^ cells/cm^2^. The 3–5 generation cells were used for the experiment. Cell growth was analyzed by direct cell count at every passage.

### Isolation and Identification of MVs

BMMSC-derived MVs were isolated using the exoEasy Maxi Kit (qiagen, Germany) according to the manufacturer's instructions. MVs were observed by Philips CM120 BioTwin transmission electron microscope (FEI, USA).

### Inhibition/Overexpression of miRNA and mRNA

miR-221-3p inhibitor, 100 nmol/L miR-221-3p mimic, 100 nmol/L overexpression of CDKN1C and the corresponding negative control (NC) were purchased from GenePharma (Shanghai, China). Approximately 1 × 10^5^ cells were inoculated into 12-well plates during transfection. CDKN1C, miR-221-3p and negative control were transfected into the cells using LipoFiter kit (Hanbio, Shanghai, China) according to the kit instructions. RNA and proteins were extracted 48 h after transfection. The sequences of synthesized primers were shown in Supplement Table 1.

### Construction of Lentivirus Expression Vector and Cell Transfection

Human miR-221-3p sequences were amplified and then bound to pcDNA3.1 (+) to form miR-221-3p expression vector (GenePharma, ShangHai, China). PcDNA3.1 carrier was used as blank control. Lentivirus coated miR-221-3p or blank lentivirus was transfected into OCI-AML2 cells and cultured for 96 h and then treated with puromycin for 4 weeks to screen cells (Santa Cruz organisms).

### qRT-PCR

Total RNA was extracted from tissues and cells using Trizol (Invitrogen) according to the manufacturer's protocol. cDNA was synthesized using reverse transcription system kit (Invitrogen). qRT-PCR was performed on ABI 7900HT instrument (Applied Biosystems, USA). Quantitative PCR was performed using the miScript SYBR Green PCR Kit (Qiagen, Germany) under the following thermal cycling conditions: pre-denaturation at 95°C for 10 min, followed by 40 cycles of denaturation at 95°C for 2 min, annealing at 95°C for 5 s and extending at 60°C for 30 s. CDKN1C was normalized with β-Actin as an internal reference, and miR-221-3p was normalized with U6 as an internal reference. The relative expression of the target gene mRNAs in the control group and the experimental group were analyzed by 2^−ΔΔCt^ method. The primers used in the experiment were shown in Supplement Table 1.

### Western Blot

Forty-eight hours after transfection of cells from different treatment groups, the cells were washed three times with cold PBS (Thermo fisher, USA), and lysed on ice using whole protein lysate for 10 min. BCA quantitative kit (Thermo fisher, USA) was used for protein quantification, then 10 μl loading buffer was added and proteins were boiled at 95°C for 10 min. the proteins were loaded onto SDS-PAGE at 100 V and transferred to the NC membrane blocked with 5% BSA/TBST for 60 min. The membrane was incubated with primary antibodies at 4°C overnight and then washed with 1 × TBST solution (Solarbio, Beijing, China) at room temperature for 5 min × 3 times. the membrane was probed with HRP labeled goat-anti-rabbit IgG at room temperature for 120 min, and washed by TBST for three times. After each 20 min, the ECL kit (Solarbio, Beijing, China) was used for detecting luminescence reaction, and the protein blot was photographed and observed. The antibodies used in experiment were listed in Supplement Table 2.

### MTT Assay

OCI-AML2 cells (5 × 10^3^ cells/100 ul) were seeded into 96-well plates. Each group was made in triplicate. Proliferation of cells were evaluated by sterile MTT solution (Beyptime) according to the instructions after culture for 12, 24, 48, and 72 h, respectively. Absorbance at 490 nm was measured using a spectrophotometer (Molecular Devices, Sunnyvale, CA, USA).

### Transwell Assays

Transwell migration assay was used to evaluate the migration ability of OCI-AML2 cells. 24-well Transwell Chambers (8 μm aperture, BD Biosciences) were used. For migration assay, cells at a density of 1 × 10^5^ cells/chamber were seeded into the upper chamber and the 600 μL of medium containing 10% FBS (Thermo fisher, USA) was placed in the lower chamber. For invasion assay, ~2 × 10^4^ cells/chamber were seeded in the upper chamber, which was coated with Matrigel. Dulbecco's modified Eagle culture medium (DMEM) containing 10% FBS (Thermo fisher, USA) was filled into the lower chamber. After incubation at 37°C for 48 h, the cells that were not migrated/invaded were cleared away with a cotton swab and the migrated/invaded cells on the lower side were stained with 0.5% crystal violet. Cells were observed under a microscope, and photographed.

### Flow Cytometry (FCM)

Cell cycle detection: OCI-AML2 cells in growth phase were added with 3 mL PBS and harvested with 1 mL trypsin for 1–5 min after removing the liquid. The cell suspension was prepared by adding 5 mL PBS, and then transferred to a 15 mL centrifuge tube for centrifugation at 1,500 rpm for 5 min to discard the supernatant. Five hundred microliters PBS was added for cell suspension, and 2 mL of cold ethanol of 95% at 20°C was added to the suspension. After mixing, the suspension was fixed for 30 min. Five milliliters PBS was added and centrifuged at 1,500 rpm for 5 min to remove the supernatant and then added with 5 mL PBS and centrifuged at 1,500 rpm for 5 min to discard the supernatant. The cells were stained with 800 μL PI at room temperature for 30 min in darkness. Cell cycle was detected by FCM.

### Dual Luciferase Assay

In order to determine the binding probability of miR-221-3p and 3′UTR of CDKN1C, a psiCHECK luciferase reporter vector (Sangon Co., LTD, ShangHai, China) was inserted into 3′UTR of CDKN1C wild type (WT) and mutated type (MUT). HEK293T cells (Thermo fisher, USA) were then inoculated in a 48-well plate and cultured for 24 h. miR-221-3p/NC and psiCHECK WT/MUT plasmids were co-transfected into cells. Finally, luciferase activity was measured by luciferase assay reagent (Promega, Fitchburg, WI, USA).

### Statistical Analysis

All data were processed by SPSS 22.0 statistical software. The measurement data were expressed as mean ± standard deviation. The comparison between the two groups was analyzed by *t*-test, in which ^*^ stood for *P* < 0.05.

## Results

### miR-221-3p Is Highly Expressed in Peripheral Blood of Patients With AML

**Figure 1:**
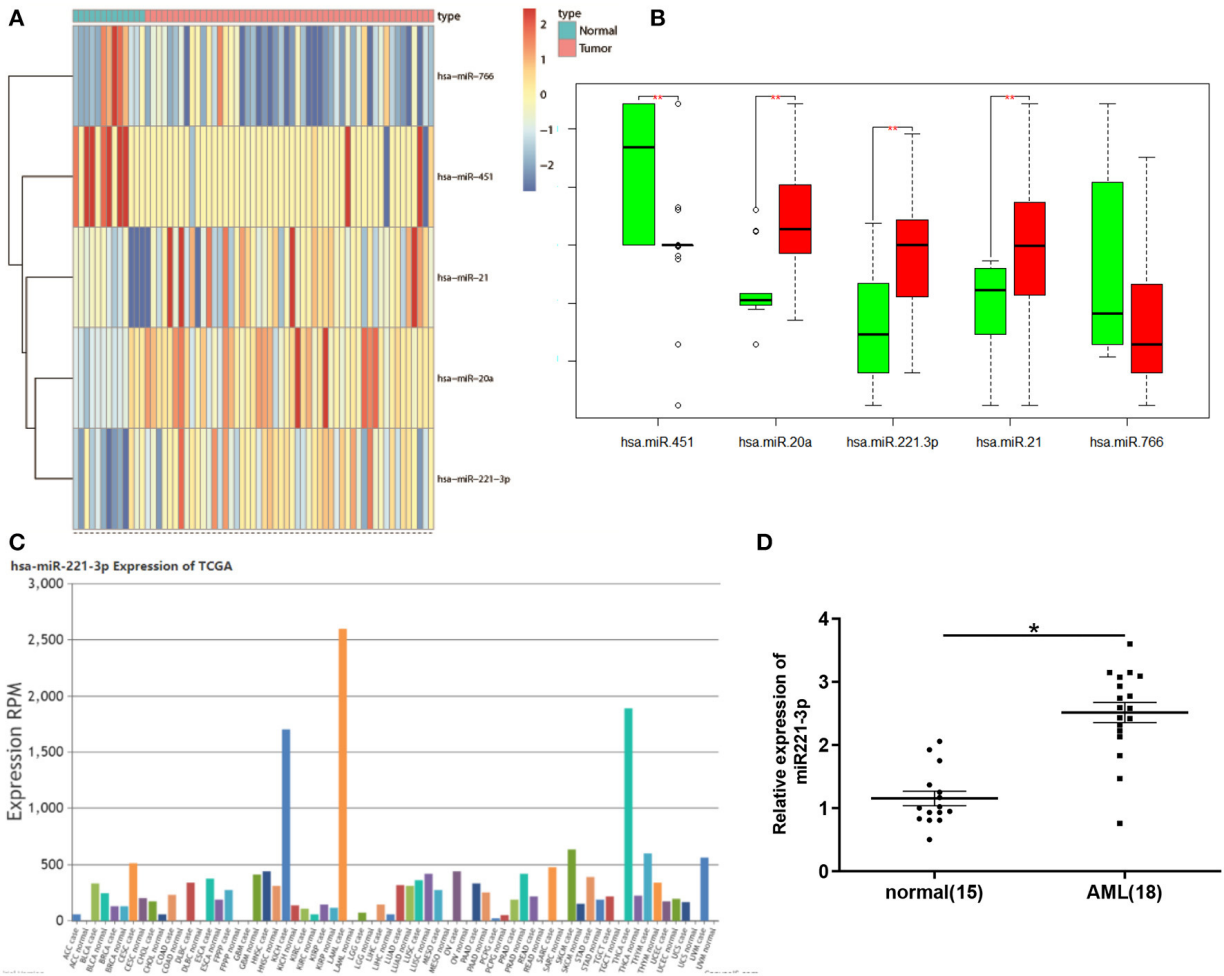
miR-221-3p is highly expressed in peripheral blood of patients with leukemia. **(A)** Heat map of DEmiRNAs in GSE49665 dataset. **(B)** Boxplot of DEmiRNA. **(C)** Expression levels of miR-221-3p in different cancer species in TCGA database. **(D)** miR-221-3p was significantly overexpressed in AML patients.

Bioinformatics analysis found that in the miRNA expression dataset GSE49665 of AML patients in the GEO database, 5 DEmiRNAs were obtained and the expression of miR-221-3p changed most significantly (Figures 1A,B). At the same time, we detected the expression level of miR-221-3p in various cancers in TCGA database and found that its expression was most significant in AML (Figure 1C), so we chose miR-221-3p for follow-up study. In order to further confirm the high expression of miR-221-3p in the peripheral blood of AML patients, we used qRT-PCR to detect the expression of miR-221-3p in the peripheral blood of 15 normal people and 18 AML patients, and discovered that miR-221-3p was highly expressed in the peripheral blood of AML patients (Figure 1D), which was consistent with the bioinformatics results.

### miR-221-3p Regulates AML Cell Cycle, Proliferation, and Invasion

**Figure 2:**
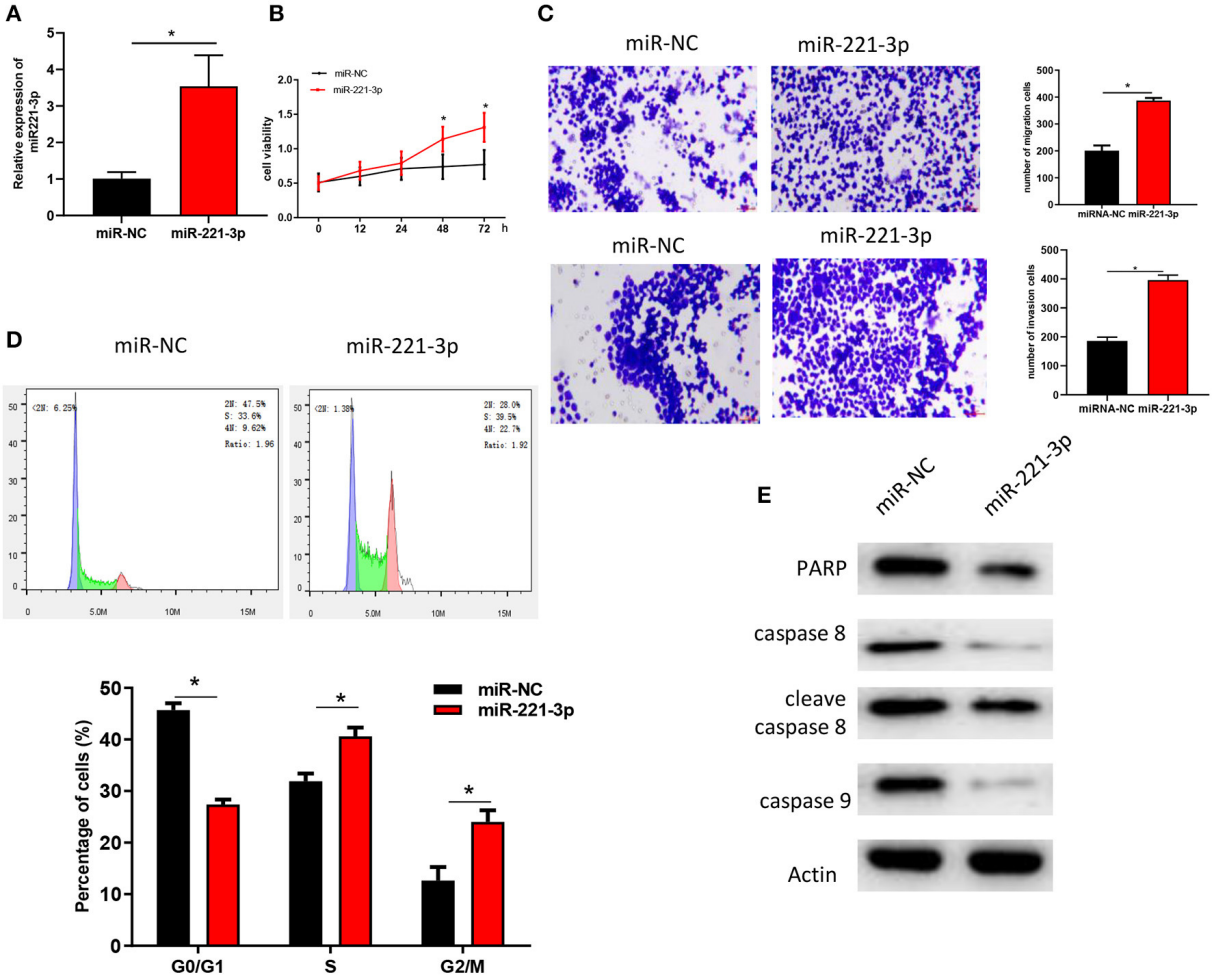
miR-221-3p regulates AML cell cycle, proliferation, and invasion. **(A)** miR-221-3p expression in each group. **(B)** The effect of miR-221-3p overexpression on the activity of OCI-AML2 cells was tested by MTT. **(C)** The effects of miR-221-3p overexpression on invasion and migration of OCI-AML2 cells were detected by Transwell assay (100×). **(D)** The effects of overexpression of miR-221-3p overexpression on the cell cycle of OCI-AML2 was detected by FCM. **(E)** The effect of miR-221-3p overexpression on expressions of apoptosis-related proteins in OCI-AML2 cells.

miR-221-3p was overexpressed in OCI-AML2 cells (Figure 2A) to further explore its role in AML. Analysis of MTT (Figure 2B) and Transwell (Figure 2C) assays revealed that overexpression of miR-221-3p could significantly improve the viability, migration and invasion abilities of OCI-AML2 cells. FCM assay were performed on NC-mimic and oe-miR-221-3p OCI-AML2 cells. The results indicated that overexpression of miR-221-3p reduced the number of cells in the G0/G1 phase with the number of cells in the divisions increased in OCI-AML2 cells (Figure 2D). The expressions of PARP, caspase 8, cleave caspase 8, caspase 9, and other apoptosis-related proteins detected by western blot were decreased after overexpression of miR-221-3p (Figure 2E), indicating that overexpression of miR-221-3p weakened the apoptosis of OCI-AML2 cells, and the results were consistent with FCM.

### BMMSC-Derived MVs Regulate the Function of AML Cells

**Figure 3:**
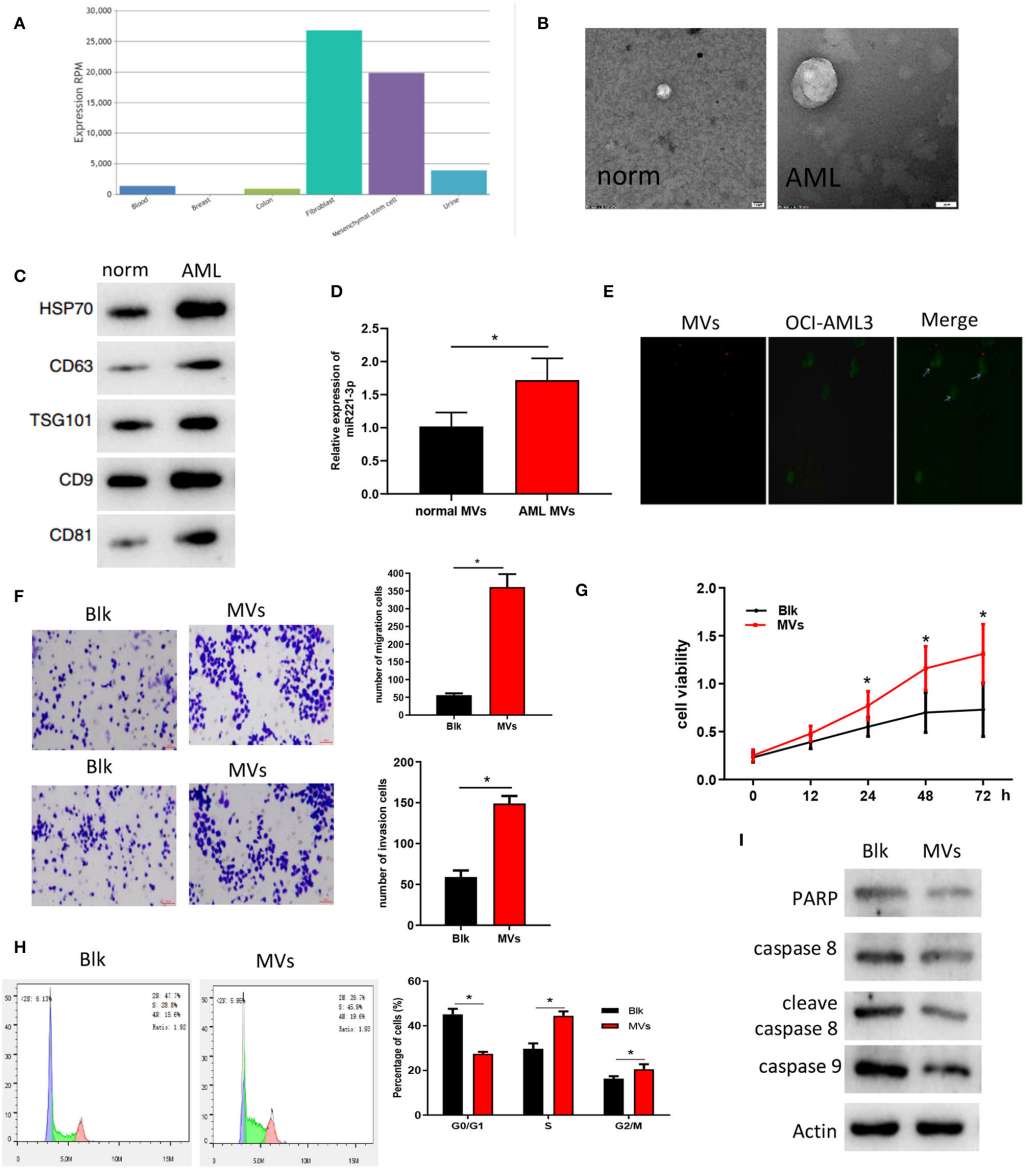
BMMSC-derived MVs regulate AML cell function. **(A)** The expression of miR-221-3p in the MVs of each cell, the abscissa represents the cell name and the ordinate represents the miRNA expression value. **(B)** The morphology and size of BMMSC-derived MVs from normal subjects and AML patients were observed under electron microscopy. **(C)** The changes of MV marker proteins from different sources were detected by western blot. **(D)** miR-221-3p expression in BMMSC-derived MVs in normal subjects and AML patients. **(E)** Fluorescence microscopy showed that Dio-labeled OCI-AML2 cells (green) were transferred to Dil-labeled MVs (red). **(F)** Transwell assay was conducted to detect the effects of MVs on the invasion and migration ability of OCI-AML2 cells (100×). **(G)** The effect of MVs on the activity of OCI-AML2 cells was determined by MTT assay. **(H)** The effects of MVs from different sources on the cell cycle of OCI-AML2 was detected by FCM. **(I)** The expression changes of apoptosis-related proteins in OCI-AML2 cells affected by MVs from different sources were determined by western blot.

At present, studies have found that miRNAs can be produced by other cells and transported to target cells through MVs for further function (Momen-Heravi et al., 2015; Hornick et al., 2016; Lu, 2017), so we speculated that miR-221-3p may be carried by MVs to act on AML cells and thus exerting its regulatory role. We searched the expression location of miR-221-3p in the EVmiRNA database and found that its content in MVs of fibroblast and MSCs was significantly higher than that in MVs of other cells (Figure 3A). Meanwhile, studies have reported that BMMSC can affect the morphology, adhesion and microenvironment of leukemia stem cells (Roversi et al., 2019). Then we hypothesized that miR-221-3p was contained in the BMMSC-derived MVs and entered the blood to affect the morphological function of AML cells. MVs of BMMSC from normal subjects and AML patients were extracted, and the morphology of MVs was observed under electron microscopy to verify the hypothesis. The MVs showed double-concave disk-like particles with a diameter of about 100 nm−1 μm (Figure 3B). The contents of MV marker proteins CD63, TSG101, HSP70, CD9, and CD81 were detected by western blot (Figure 3C) to verify the successful extraction of MVs. Further detection revealed that the expression of miR-221-3p in the BMMSC-derived MVs of AML patients was significantly higher than that of normal subjects (Figure 3D). To identify the delivery of MVs, we labeled BMMSC-derived MVs and OCI-AML2 cells with Dil (red) or Dio (green), respectively. After co-culture, it was observed that Dil spots presented in the OCI-AML2 cells under laser scanning confocal microscope, indicating that the MVs released by the BMMSC were delivered to the OCI-AML2 cells (Figure 3E). Finally, the results of Transwell (Figure 3F) and MTT (Figure 3G) assays showed that the BMMSC-derived MVs significantly improved the migration and invasion abilities as well as cellular activity of OCI-AML2 cells. The results of FCM on co-cultured cells showed that the number of cells was reduced in G0/G1 phase and increased in division stage of the OCI-AML2 cell cycle induced by BMMSC-derived MCs (Figure 3H). The expressions of apoptosis-related proteins including PARP, caspase 8, cleave caspase 8, and caspase 9 determined by western blot were decreased after the co-culture of BMMSC-derived MVs with OCI-AML2 cells (Figure 3I), indicating that the MVs could reduce the apoptosis of OCI-AML2 cells, which was in keeping with the results of FCM. These results demonstrated that the BMMSC-derived MVs could enter OCI-AML2 cells, promote the proliferation, migration and invasion, weaken the apoptosis and regulate the cell cycle of OCI-AML2 cells.

### BMMSC-Derived MVs Regulates Cell Biological Behaviors in AML via miR-221-3p

**Figure 4:**
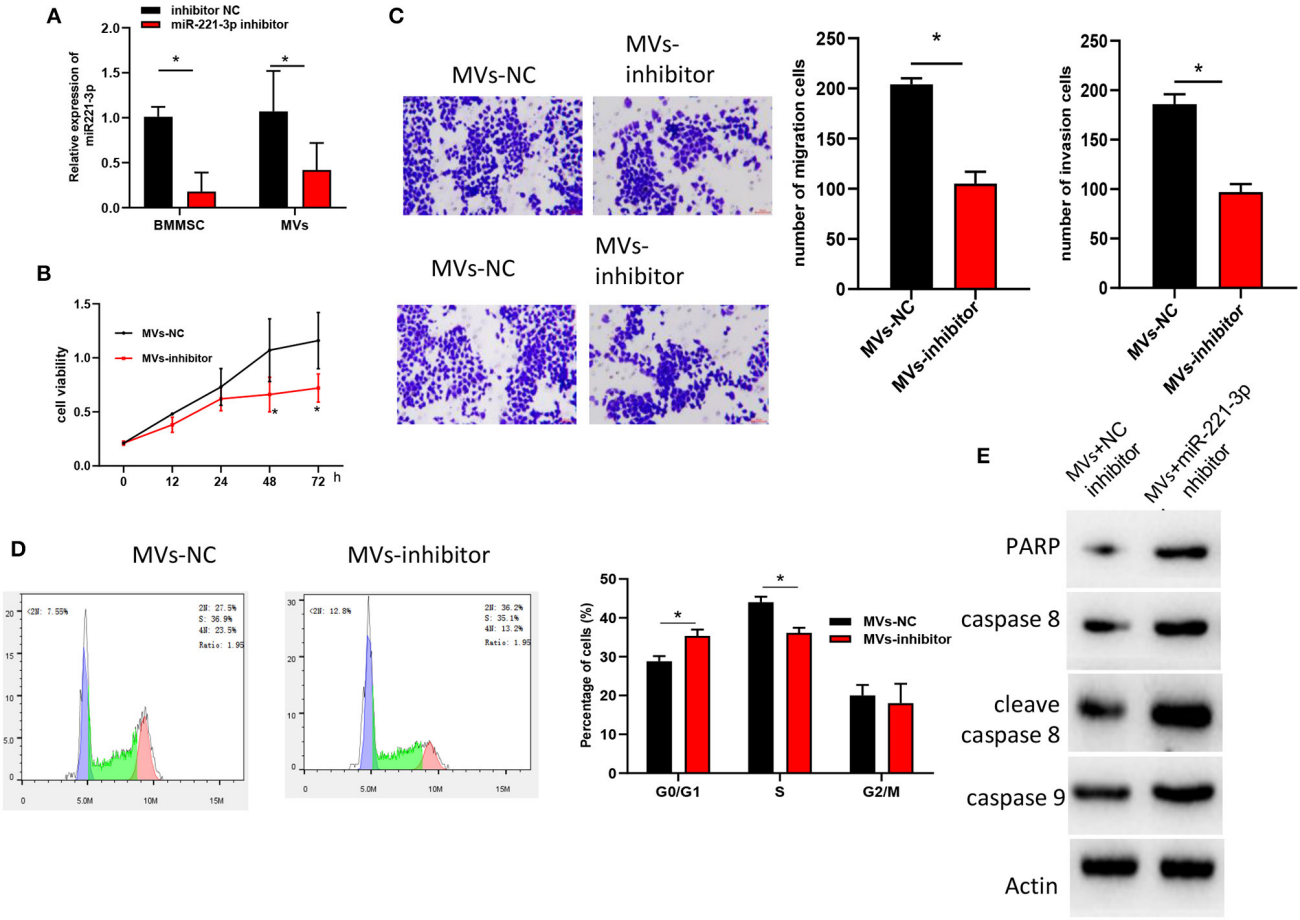
BMMSC-derived MVs regulates cell biological behaviors in AML via miR-221-3p. **(A)** miR-221-3p expression in BMMSC and MVs. **(B)** OCI-AML2 cell viability detected by MTT. **(C)** Cell migration and invasion assayed by Transwell (100×). **(D)** Cell cycle and cell apoptosis test by FCM. **(E)** Levels of apoptosis-related proteins determined by western blot.

To further investigate the regulatory mechanism of BMMSC-derived MVs on OCI-AML2 cell proliferation, invasion and cell cycle via miR-221-3p, inhibitor NC and miR-221-3p inhibitor were transfected into BMMSC, respectively. MVs in two groups were extracted and we found that miR-221-3p was significantly decreased in MVs with miR-221-3p inhibitor relative to that in MVs with NC inhibitor (Figure 4A). Then, the MVs were co-cultured with OCI-AML2 cells, showing that MVs in miR-221-3p inhibitor group suppressed the promotive effect of BMMSC-derived MVs on cell proliferation, migration and invasion of OCI-AML cells (Figures 4B,C). Meanwhile, FCM revealed that miR-221-3p inhibitor induced BMMSC cell cycle arrested in G0/G1 phase, indicating that miR-221-3p inhibitor could reverse the effect of BMMSC-derived MVs on cell cycle (Figure 4D). Besides, high expressions of apoptosis-related proteins PARP, caspase 8, cleave caspase 8, and caspase 9 were detected by western blot (Figure 4E), and the results suggested that miR-221-3p was capable of abrogating the inhibitory effect of BMMSC-derived MVs on OCI-AML2 cells, which was consistent with the FCM results. In all, these findings shed light on that BMMCS-derived MVs regulated OCI-AML2 cell biological behaviors via miR-221-3p.

### miR-221-3p Regulates Cell Proliferation, Invasion and Cell Cycle in AML via Targeting CDKN1C

**Figure 5:**
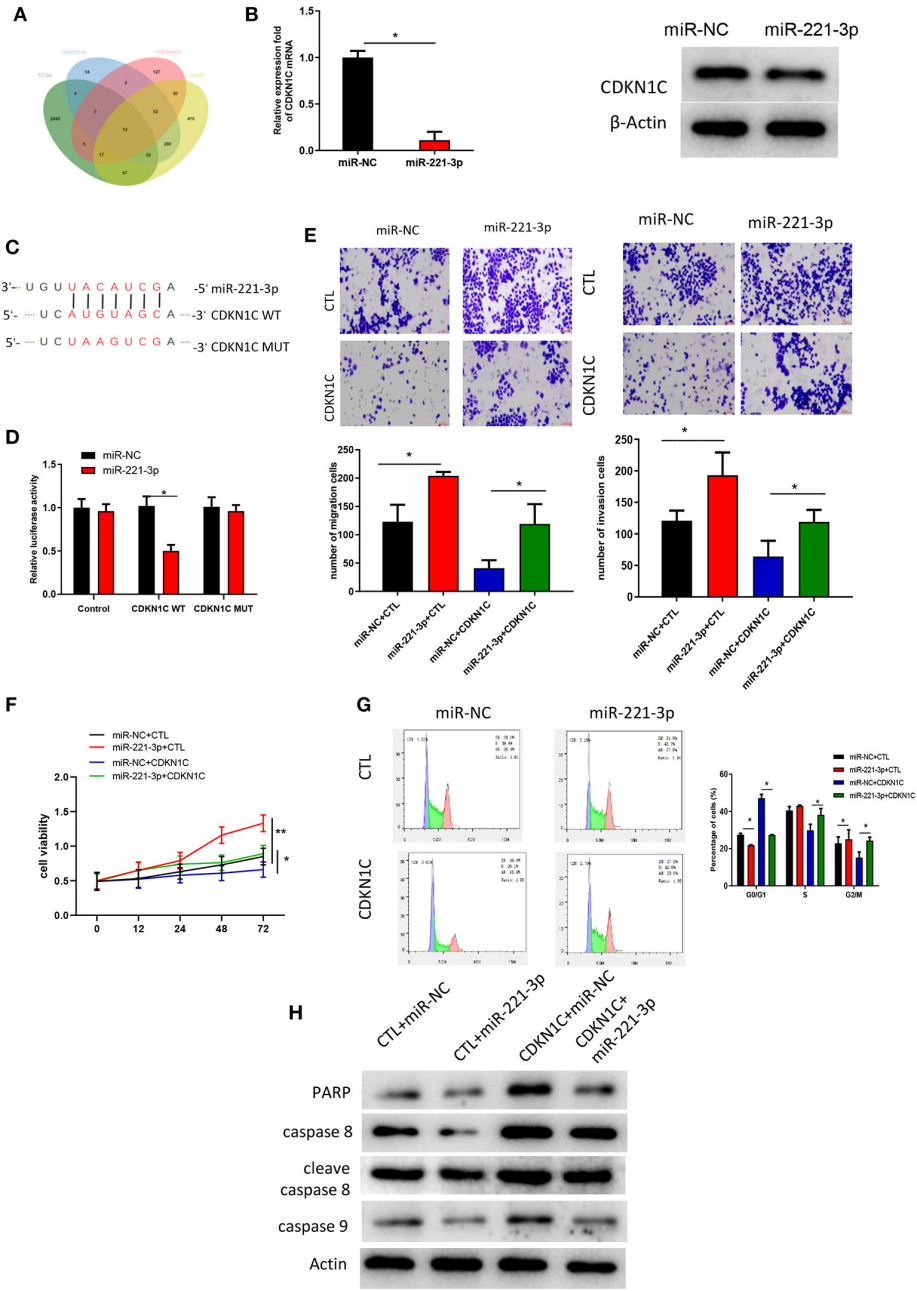
miR-221-3p regulates cell proliferation, invasion and cell cycle in AML via targeting CDKN1C. **(A)** Venn daigram was plotted to find the potential target genes of miR-221-3p. **(B)** CDKN1C expression in mRNA and protein levels in the presence of miR-221-3p overexpression. **(C)** The binding sites of miR-221-3p on CDKN1C 3′-UTR-WT and CDKN1C 3′-UTR-MUT. **(D)** Relative luciferase activity in each group. **(E)** Cell migration and invasion detected by Transwell (100×). **(F)** Cell viability test by MTT. **(G)** Cell cycle determined by FCM. **(H)** Protein levels of apoptosis-related proteins measured by western blot.

The downstream targets of miR-221-3p were predicted by TargetScan, miRSearch and mirDIP databases. Differential analysis was performed on the mRNAs procured from TCGA-AML dataset, and eventually 12 potential targets were obtained after the intersection between the identified down-regulated DEmRNAs and the predicted targets (Figure 5A). Among the 12 target genes, CDKN1C alteration in AML was shown to be the most significant (Table 1). Thereafter, to further validate the relationship between miR-221-3p and CDKN1C, miR-221-3p mimic and NC-mimic were, respectively, transfected into OCI-AML2 cells. Western blot and qRT-PCR suggested that the mRNA and protein expressions CDKN1C were reduced in the cells transfected with miR-221-3p mimic (Figure 5B). Then, online miRNA data analysis software (starBase) was applied, finding that miR-221-3p was targeted binding with the CDKN1C 3′-UTR (Figure 5C). Meanwhile, dual-luciferase assay demonstrated that miR-221-3p inhibitory functioned on the luciferase activity in cells transfected with CDKN1C-WT, whereas there was no difference observed in cells transfected with CDKN1C-MUT (Figure 5D). Taken together, we could conclude that CDKN1C was a direct target of miR-221-3p. Subsequently, a series of *in vitro* experiments were conducted to explore the miR-221-3p-dependent mechanism on cell biological behaviors via CDKN1C. Transwell and MTT assays showed that overexpressing of CDKN1C could reverse the promotive role of miR-221-3p overexpression in cell migration, invasion, proliferation and colony forming of OCI-AML cells (Figures 5E,F). Besides, the effects of miR-221-3p overexpression on cell cycle could also be reversed when CDKN1C was simultaneously increased (Figure 5G). Moreover, apoptosis-related proteins were all observed to be elevated after CDKN1C being overexpressed (Figure 5H), elucidating that CDKN1C overexpression was capable of rescuing the decrease of cell apoptosis induced by miR-221-3p overexpression.

**Table 1:** Differential expression of the identified 12 potential target genes in the TCGA-AML dataset.

**Gene symbol**	**Gene ID**	**Median (Tumor)**	**Median (Normal)**	**Log2 (Fold Change)**	**Adjp**
CDKN1C	ENSG00000129757.12	13.44	118.61	−3.05	4.88E-22
NRK	ENSG00000123572.16	0.93	9.153	−2.395	1.77E-10
MYLIP	ENSG00000007944.14	2.83	13.58	−1.929	8.84E-26
POGZ	ENSG00000143442.21	11.19	31.24	−1.403	1.20E-27
FOS	ENSG00000170345.9	28.099	75.439	−1.393	3.10E-09
ARHGEF7	ENSG00000102606.17	9.05	23.855	−1.306	4.83E-36
CREBZF	ENSG00000137504.13	13.06	32.565	−1.255	2.01E-27
FAM214A	ENSG00000047346.12	12.69	30.505	−1.202	6.79E-18
ADAM22	ENSG00000008277.14	1.37	4.23	−1.142	2.88E-14
CD4	ENSG00000010610.9	3.35	8.185	−1.078	2.74E-07
HMBOX1	ENSG00000147421.17	14.57	31.545	−1.064	1.92E-21
RFX7	ENSG00000181827.14	1.66	4.535	−1.057	3.91E-26

## Discussion

MVs were primarily regarded as unfunctional cellular components to be discarded, yet it has been increasingly suggested that MVs are important tools for the exchange of cellular information and materials, and closely correlated with tumor distant metastasis and immune inhibition (Steinbichler et al., 2017; Fan et al., 2018; Seo et al., 2018; Jerez et al., 2019). MVs are capable of inducing various biological processes after being transferred into recipient cells, such as angiogenesis, metastasis formation, therapeutic resistance, epithelial-mesenchymal transition (EMT) and epigenetic programming (Kreimer et al., 2015; Milane et al., 2015; Gopal et al., 2017). In the present study, we found that miR-221-3p was highly expressed in BMMSC-derived MVs. Besides, it has been reported that bone marrow stromal cell-derived MVs can attenuate the B cell apoptosis in chronic lymphocytic leukemia, also promote cell migration and induce gene expression and modification (Crompot et al., 2017). Hence, this study focused attention on the BMMSC MVs-derived miR-221-3p. Enormous studies have revealed that miR-221-3p is aberrantly expressed in various cancers and participate in the regulation of tumorigenesis and development, like cervical squamous carcinoma (Wu et al., 2019), hepatocellular carcinoma (Li et al., 2019), medulloblastoma (Yang et al., 2019), and breast cancer (Ergun et al., 2015). However, the role of miR-221-3p in AML has not been reported. Therefore, the purpose of this study is to explore the mechanism of miR-221-3p in AML. In our study, we discovered that miR-221-3p was mainly present in BMMSC-derived MVs, and found to be overexpressed in AML patients. Then we constructed miR-221-3p overexpression and found that elevated miR-221-3p was responsible for the promotion of OCI-AML2 cell proliferation, migration and invasion. Moreover, miR-221-3p has been reported to play an important role in other cancers. Wu et al. have found that miR-221-3p from tumor cell-derived MVs targets THBS2 to facilitate the angiogenesis in cervical squamous carcinoma (Wu et al., 2019). Wei et al. have reported that miR-221-3p can potentiate metastasis in cervical cancer via directly targeting THBS2 (Wei et al., 2017). In addition, Shi et al. have revealed that miR-221-3p serving as an oncogene promotively functions on cell proliferation, migration and invasion in gastric cancer through inhibiting PTEN (Shi et al., 2017). Collectively, we believed that miR-221-3p from BMMSC-derived MVs could act as an oncogene beneficial for the cell proliferation, migration and invasion in AML.

In order to further understand the molecular mechanism of miR-221-3p regulating the function of AML cells in BMMSC-derived MVs, we proved that miR-221-3p can directly target CDKN1C through bioinformatics analysis and dual-luciferase assay. Besides, there was a negative correlation showed in miR-221-3p and CDKN1C expressions both in tissues and cells. CDKN1C is a cyclin-dependent kinase inhibitor 1C, which can inhibit cell proliferation (Adkins and Lumb, 2002; Qiu et al., 2018, 2019). Abnormal expression of CDKN1C plays a role in breast cancer (Qiu et al., 2018), gastric cancer (Sun et al., 2017), glioma (Zhang et al., 2015) and other cancers. And some studies have found that CDKN1C is often methylated in acute lymphoblastic leukemia, and methylation is associated with poor prognosis (Shen et al., 2003). It is found that the expression of CDKN1C is related to the prognosis of patients with AML (Radujkovic et al., 2016), but the biological function of CDKN1C in AML is unclear. This study found that overexpressing CDKN1C could suppress cell proliferation, migration and invasion in AML. Moreover, CDKN1C was able to reverse the regulation of miR-221-3p overexpression on AML cell biological behaviors when it was concurrently elevated. Taken together, these results suggest that miR-221-3p in BMMSC-derived MVs in AML patients regulates the proliferation, invasion, migration and cell cycle by targeting CDKN1C.

In conclusion, our study confirmed that miR-221-3p from BMMSC-derived MVs had the functions of promoting cell proliferation, migration, invasion and regulating cell cycle in AML via targeting CDKN1C. This finding extends our knowledge on the role of miR-221-3p in AML, and helps to further explore the novel approaches for AML targeted therapy.

## Data Availability Statement

The data used to support the findings of this study are included within the article. The data and materials in the current study are available from the corresponding author on reasonable request.

## Author Contributions

XuZ and WY contributed to the study design. YX, JW, SZ, and JL conducted the literature search. XH and HX acquired the data. XiZ and SS wrote the article. YL performed data analysis and drafted. YZ and YX revised the article.

### Conflict of Interest

The authors declare that the research was conducted in the absence of any commercial or financial relationships that could be construed as a potential conflict of interest.
