# Supplementary material for: miR-221-3p Delivered by BMMSC-Derived Microvesicles Promotes the Development of Acute Myelocytic Leukemia
Source: Front Bioeng Biotechnol. 2020 Feb 14;8:81. doi: 10.3389/fbioe.2020.00081 (PMC7033425; doi:10.3389/fbioe.2020.00081)
Supplement: Supplementary file 1 [file Table_1.DOCX]

**Supplement table 1 The sequence of primers used in the experiment**

| **qPCR** | **sense sequence****（5’-3’）** | **anti-sense sequence（5’-3’）** |
| --- | --- | --- |
| miR-221-3p | TCGGCAGGAGCTACATTGTCTGC | CTCAACTGGTGTCGTGGA |
| CDKN1C | ATCCACGATGGAGCGTCTTG | TTGCAGCATTTTTCGGCCTC |
| U6 | CTCGCTTCGGCAGCACA | AACGCTTCACGAATTTGCGT |
| GAPDH | ACAACTTTGGTATCGTGGAAGG | GCCATCACGCCACAGTTTC |
