# Supplementary material for: miR-221-3p Delivered by BMMSC-Derived Microvesicles Promotes the Development of Acute Myelocytic Leukemia
Source: Front Bioeng Biotechnol. 2020 Feb 14;8:81. doi: 10.3389/fbioe.2020.00081 (PMC7033425; doi:10.3389/fbioe.2020.00081)
Supplement: Supplementary file 2 [file Table_2.DOCX]

**Supplement table 2 Antibodies used in the laboratory**

| **Antibody** | **WB** | **Specificity** | **Company** |
| --- | --- | --- | --- |
| β-actin | 1:1000 | Rabbit monoclonal | Abcam, China |
| p57 Kip2  (CDKN1C) | 1:1000 | Rabbit monoclonal | Abcam, China |
| HSP70 | 1:1000 | Rabbit monoclonal | Abcam, China |
| CD63 | 1:1000 | Rabbit polyclonal | Abcam, China |
| TSG101 | 1:1000 | Rabbit monoclonal | Abcam, China |
| CD9 | 1:2000 | Rabbit monoclonal | Abcam, China |
| CD81 | 1:1000 | Rabbit monoclonal | Abcam, China |
| PARP | 1:1000 | Rabbit monoclonal | Abcam, China |
| caspase 8 | 1:1000 | Rabbit monoclonal | Abcam, China |
| Cleave caspase 8 | 1:1000 | Rabbit monoclonal | CST, USA |
| caspase 9 | 1:2000 | Rabbit monoclonal | Abcam, China |
| **Antibody** | **IHC** | **Specificity** | **Company** |
| Ki67 | 1:1000 | Rabbit monoclonal | Abcam, China |
